# Unsaturated long-chain fatty acids induce the respiratory burst of human neutrophils and monocytes in whole blood

**DOI:** 10.1186/1743-7075-5-19

**Published:** 2008-07-14

**Authors:** Björn Jüttner, Janina Kröplin, Sina M Coldewey, Lars Witt, Wilhelm A Osthaus, Christian Weilbach, Dirk Scheinichen

**Affiliations:** 1 Department of Anaesthesiology, Hannover Medical School, Germany; 2 Department of Anaesthesiology, St. Josefs-Hospital, Cloppenburg, Germany

## Abstract

**Background:**

It is increasingly recognized that infectious complications in patients treated with total parenteral nutrition (TPN) may be caused by altered immune responses. Neutrophils and monocytes are the first line of defence against bacterial and fungal infection through superoxide anion production during the respiratory burst. To characterize the impact of three different types of lipid solutions that are applied as part of TPN formulations, we investigated the unstimulated respiratory burst activation of neutrophils and monocytes in whole blood.

**Methods:**

Whole blood samples were incubated with LCT (Intralipid^®^), LCT/MCT (Lipofundin^®^) and LCT-MUFA (ClinOleic^®^) in three concentrations (0.06, 0.3 and 0.6 mg ml^-1^) for time periods up to one hour. Hydrogen peroxide production during the respiratory burst of neutrophils and monocytes was measured by flow cytometry.

**Results:**

LCT and LCT-MUFA induced a hydrogen peroxide production in neutrophils and monocytes without presence of a physiological stimulus in contrast to LCT/MCT.

**Conclusion:**

We concluded that parenteral nutrition containing unsaturated oleic (C18:1) and linoleic (C18:2) acid can induce respiratory burst of neutrophils and monocytes, resulting in an elevated risk of tissue damage by the uncontrolled production of reactive oxygen species. Contradictory observations reported in previous studies may in part be the result of different methods used to determine hydrogen peroxide production.

## Background

Intravenous fat emulsions are an integral part of total parenteral nutrition. However, for a variety of parenteral fat emulsions, significant impact on the immune response has been reported [1]. Parenteral nutrition has been associated with an increased complication rate due to infections [2]. Immune-suppressive effects of the lipid component have been suggested to be at least partially responsible for this complication [3].

The type of infections encountered in patients treated with total parenteral nutrition suggests that especially the function of the polymorphonuclear leukocytes (neutrophils) is compromised. Neutrophils protect the human body from microbial invaders by phagocytosis followed by destruction through the production of superoxide anions, the so called respiratory burst. Circulating monocytes are immune cells also involved in phagocytosis, furthermore in antigen presentation and cytokine production.

Experimental studies regarding the effects of lipid emulsion on neutrophils and monocyte-macrophages function are controversial [4].

Arachidonic acid (C20:4ω-6) appears to be important in neutrophils as an activator of the respiratory burst [5]. Otherwise, most reports indicate inhibitory or no effects on these cells by long-chain triglyceride (LCT) emulsions, which are particularly rich in ω-6 polyunsaturated fatty acids (PUFAs) [4].

In vitro studies suggest that emulsions containing a mixture of ω-6 long-chain and medium-chain triglycerides (LCT/MCT) induce an increased oxygen radical production and the expression of adhesion and degranulation markers in neutrophils [6-8].

In contrast, other investigators found that stimulated neutrophilic oxygen radical production was uniformly inhibited by a variety of emulsions containing LCT, MCT/LCT or structured lipids (SL) [9]. Furthermore, in vivo no detrimented effects have been reported for MCT [10,11].

Nearly all of the information regarding lipid effects on circulating phagocytes comes from experimental studies in which leukocytes were isolated from whole blood prior to lipid incubation. A leukocyte isolation procedure by density gradient centrifugation seems to be able to introduce functional changes, especially pre-activation [12,13]. In an in vitro study whole blood incubation was used for the first time to analyse leukocytes following lipid exposure [14].

This study was designed to examine the impact of three structurally different types of lipid solutions that are applied as a part of total parental nutrition, i.e. LCT (Intralipid^®^), LCT/MCT (Lipofundin^®^) and LCT-MUFA (ClinOleic^®^) on the unstimulated hydrogen peroxide production of neutrophils and monocytes in whole blood.

## Methods

This study was performed with the approval of the local ethic committee and the informed consent of the 12 male and 8 female healthy volunteers, aged 38.25 ± 12.27 years.

Venous blood samples (5 ml) were collected into disposable tubes coated with lithium heparin (15 IU ml^-1^, S-Monovette LH, Sarstedt, Nümbrecht, Germany). The volunteers were non-smokers and not vegetarian. They did not take any medication or dietary supplements and fasted the night before sample collection. All samples were taken, processed and measured in duplicate by the same investigator.

The effects of the tested lipid emulsions on the respiratory burst were measured by the intracellular oxidation of dihydrorhodamine (DHR) to the fluorescent dye rhodamine 123 in a flow cytometer [15,16]. The assay depends upon the incorporation of DHR into the cell. After cell activation the nicotinamide adenine dinucleotide phosphate (NADPH) oxidase catalyses the reduction of O_2 _to superoxide anion O_2_^- ^which is further transformed by dismutation to hydrogen peroxide H_2_O_2_. The non-fluorescent DHR is oxidized intracellularly in a peroxidase-dependent reaction to green fluorescent rhodamine. The amount of rhodamine is proportional to generated hydrogen peroxide.

Thirty microliters (μl) DHR (0.11 mM, Sigma, Deisenhofen, Germany) were added to tubes containing 100 μl heparinized whole blood. The lipid emulsions LCT (Intralipid^® ^20%, Baxter, Unterschleißheim, Germany), LCT/MCT (Lipofundin MCT^® ^20%, Braun, Melsungen, Germany) and LCT-MUFA (ClinOleic^® ^20%, Baxter, Unterschleißheim, Germany) were added to three tubes with identical volumes and concentrations of 0.06 mg ml^-1^, 0.3 mg ml^-1 ^and 0.6 mg ml^-1 ^(a concentration of 0.6 mg ml^-1 ^is equivalent to 0.69 mM LCT, 0.95 mM LCT/MCT and 0.69 mM LCT-MUFA respectively). Respective composition of the used lipid emulsions are presented in Table 1 (see additional file 1). Tubes were incubated at 37°C for up to 1 hour and samples were taken every 10 min. The reactions were stopped by the addition of 100 μl OptiLyse (Immunotech, Krefeld, Germany), a reagent that lyses erythrocytes and fixes leukocytes. Samples were allowed to cool to room temperature. After fifteen minutes, cells were collected by centrifugation and resuspended in 500 μl phosphate buffered saline (PBS). DNA staining of permeabilized leukocytes with 50 μl propidium iodide (PI, 0.15 mM, Sigma) was performed to exclude aggregation artefacts. Samples were analysed in a flow cytometer within 30 min (EPICS XL, Coulter, Krefeld, Germany).

Whole blood with an equal volume of PBS instead of lipid solutions was used as negative control. For the positive control the respiratory burst was induced by addition of 20 μl *E. coli *(3 × 10^7 ^ml^-1^, strain HB 101) to whole blood, without addition of lipid solutions.

### Measurement

**Figure 1:**
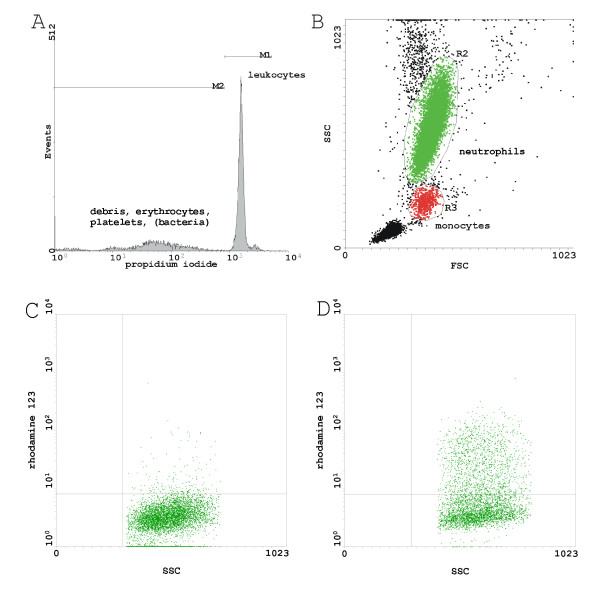
**Flow cytometry analysis**. (A) Live gate on leukocyte DNA (M1) during acquisition: Discrimination of leukocytes from debris, erythrocytes, platelets and bacteria. (B) Neutrophils and monocytes were identified by setting a polygonal gate in a forward scatter/sideward scatter dot plot. The negative control sample (C) was used to define a marker for rhodamine 123 (FL 1) where less than 5% of the cells would be positive. The percentage of neutrophils having produced hydrogen peroxide following lipid incubation was determined by counting the number of rhodamine positive cells above this marker position and by dividing it by the whole number of events observed (D).

Cells were analyzed by flow cytometry using the blue-green excitation light (488 nm argon-ion laser). Twenty thousand events were included for each measurement. According to the method described by Hirt et al. [17] a linear region on the PI peak signal in the histogram of fluorescence (FL) 3 was set to discriminate between nuclear (i.e. leukocytes) and non-nuclear cells (debrids, erythrocytes, platelets) and between human diploid and haploid cells (bacteria), respectively, Figure 1A. Neutrophils and monocytes were identified by setting a polygonal gate in a forward scatter/sideward scatter dot plot, Figure 1B. The negative control sample was used to define a marker for rhodamine (FL 1) where less than 5% of the events would be positive, Figure 1C. The percentage of cells having produced hydrogen peroxide was determined by counting the number of rhodamine positive cells above this marker position and by dividing it by the whole number of events observed, Figure 1D.

In addition, as we described recently [18], respiratory burst activity was determined with the content of rhodamine (FL 1) in the phagocytic cells, expressed as mean fluorescence intensity of the FL 1 histogram.

### Calculations and Statistics

All numeric data showed a Gaussian distribution (Kolmogorov-Smirnov test). A univariate multifactorial analysis of variance (ANOVA) was used to identify intergroup effects of different lipid emulsions, concentrations and time (SPSS/PC^® ^V 14.0 software package, SPSS, Munich, Germany). Post hoc univariate one factor ANOVA was used to compare lipid emulsions, concentrations and controls, followed by the Dunnett-t-test. A probability of less than 0.05 was considered significant. In all experiments, the data was expressed as the mean ± standard error of mean (SEM).

## Results

In the present study the percentage of neutrophils and monocytes having produced hydrogen peroxide was analysed by flow cytometry after incubation with three different lipid emulsions for up to 1 hour as described above. The positive control stimulated by *E. coli *showed an adequate increase of cells having produced hydrogen peroxide in all samples (neutrophils 77.4 ± 3.0 %; monocytes 52.3 ± 2.9 %).

We found a non significant increase of the spontaneous hydrogen peroxide production in both phagocyting cell populations over the incubation period of 1 h (neutrophils from 5.3 ± 1.3 % to 8.1 ± 1.1 %, monocytes from 3.6 ± 1.1 % to 5.8 ± 1.2 %). Furthermore, comparing the different lipid groups by multifactorial analysis, we observed no statistically significant time dependency.

**Figure 2:**
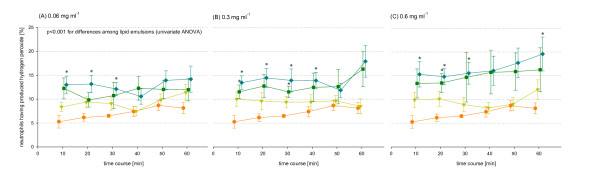
**Time course of neutrophils having produced hydrogen peroxide**. (●) control without lipid emulsions and incubation with (A) 0.06 mg ml^-1^, (B) 0.3 mg ml^-1 ^and (C) 0.6 mg ml^-1 ^of (▼) LCT/MCT, (■) LCT-MUFA and (◆) LCT. *p < 0.05 lipid emulsion versus negative control, post hoc Dunnett-t-test.

Alterations of the hydrogen peroxide production of phagocytes depended on the lipid group and on the concentration of the lipid emulsion, Figure 1. Exposure to LCT/MCT resulted in no significant differences of neutrophil and monocyte hydrogen peroxide production compared to negative control values, Figure 2, Table 2 and 3 (see additional file 1). Incubation with LCT-MUFA or LCT was associated with significant changes in the rhodamine fluorescence signal and side scatter characteristics of a subpopulation of neutrophils.

### Percentage of neutrophils and monocytes having produced hydrogen peroxide

Comparing all data of the observed time period, there was a highly significant difference among lipid emulsions for each concentration used in the present study (p < 0.001), Figure 2. In detail, incubation with LCT at a concentration of 0.06 mg ml^-1 ^lead to a significant (p < 0.05) increase of percentage of neutrophils having produced hydrogen peroxide compared to negative controls after 10, 20 and 30 min. LCT concentrations of 0.3 mg ml^-1 ^and 0.6 mg ml^-1 ^significantly increased neutrophils having produced hydrogen peroxide after 10, 20, 30 and 40 min and after 10, 20, 30 and 60 min respectively, Table 2 and Figure 2. In monocytes we observed a significant increase of cells having produced hydrogen peroxide most after incubation with LCT at the concentration of 0.6 mg ml^-1 ^compared to negative control values, Table 3. Furthermore, in monocytes there was a significant difference of the effects of LCT compared to LCT/MCT after 20, 30 and 40 min at a concentration of 0.6 mg ml^-1^.

Addition of LCT-MUFA resulted in significantly increased hydrogen peroxide production of neutrophils after 10 and 30 min at a concentration of 0.3 mg ml^-1 ^and after 20 min at a concentration of 0.6 mg ml^-1 ^compared to negative control values.

For neutrophils univariate one factor ANOVA showed a significant intragroup difference between LCT concentration of 0.6 mg ml^-1 ^and 0.06 mg ml^-1 ^(p = 0.024). Similarly, for monocytes we identified differences between LCT concentrations of 0.6 mg ml^-1 ^and 0.3 mg ml^-1 ^(p < 0.001) and between 0.6 mg ml^-1 ^and 0.06 mg ml^-1 ^(p < 0.001). Monocytes having produced hydrogen peroxide after LCT-MUFA incubation with 0.6 mg ml^-1 ^were also different from an incubation with 0.06 mg ml^-1 ^LCT-MUFA (p = 0.022).

### Mean fluorescence activity of neutrophils and monocytes

Taking all data of the observed time period into consideration, there was a significant difference among lipid emulsions in neutrophils incubated with 0.06 mg ml^-1 ^(p = 0.048), 0.3 mg ml^-1 ^(p = 0.001) and 0.6 mg ml^-1 ^(p < 0.001). The mean fluorescence activity of monocytes was statistically different among lipid emulsions incubated at a concentration of 0.6 mg ml^-1 ^(p = 0.008).

Subsequent statistically post hoc analyses revealed significant differences following LCT incubation of 0.6 mg ml^-1^. Mean fluorescence activity of neutrophils was different to negative control values after 10 min (42.42 ± 3.13 vs. 30.53 ± 2.32, lipid incubation vs. control) and 30 min (42.35 ± vs. 31.85 ± 0.44). Monocytes differ to negative control after 30 min (29.77 ± 1.69 vs. 24.18 ± 0.11) incubation.

## Discussion

Total parenteral nutrition is a well established and safe therapy used to supply all essential nutrients. Nevertheless, it has been clearly shown that lipid components are associated with a higher risk for complications due to infections [2]. Various studies have described an alteration especially of the unspecific immune response, dependent on the composition of fatty acids used [19]. The number of double bonds, the position of the double bonds and the carbon chain length of the fatty acid molecules seemed to be critical factors.

Neutrophils and monocytes have key functions in the immune system, particularly in killing microbial invaders and the regulation of the inflammatory response. Therefore, we investigated in a close to physiological whole blood technique the effect of three structurally different lipid emulsions on the unstimulated hydrogen peroxide production of neutrophils and monocytes. We found a recognized certain variability in measurement of hydrogen peroxide production. In accordance, Lun et al. [20] demonstrated a high interindividual variation in neutrophils respiratory burst measured by flow cytometry in all age groups. Thus, we have focused on cells from healthy volunteers because results in patients are invariably influenced by the underlying disease.

Our data shows that LCT and to a lesser extent LCT-MUFA induced neutrophils and monocytes having produced hydrogen peroxide in a dose-dependent manner, Figure 2. We hypothesized that the percentage of cells having produced hydrogen peroxide following lipid incubation represents phenotypical changes of a subpopulation in neutrophils and monocytes, Figure 1. However, no significant increased hydrogen peroxide production was found after incubation with LCT/MCT during the entire observation period.

The lipid concentrations used in our study were chosen on the basis of the results previously described by Bellinati-Pires and colleagues [21] who demonstrated that MCT-containing emulsions stimulated the oxidative metabolism of neutrophils measured with nitroblue tetrazolium (NBT) reduction. In that study, the stimulatory effect of MCT on the ability of neutrophils to reduce NBT was found to be dose dependent. The authors investigated the effects of several MCT concentrations (between 0.04 and 20 mg ml^-1^) on the percentage of neutrophils capable of NBT reduction. They found that at a MCT concentration of 0.32 mg ml^-1 ^approximately 40 % of neutrophils reduced NBT. Another group compared the effects of different lipid emulsions from fish oil, LCT-MUFA, LCT and LCT/MCT on neutrophil activation in vitro [22]. Half-maximal inhibition was reached at emulsion concentrations of 0.24 mM fish oil, 0.32 mM LCT/MCT, 0.52 mM LCT and 0.82 mM LCT-MUFA. In consideration of these results, the concentrations used in our study (the concentration of 0.6 mg ml^-1 ^being equivalent to 0.69 mM LCT, 0.95 mM LCT/MCT and 0.69 mM LCT-MUFA) appeared adequate and comparable. Also, the investigated lipid dose almost corresponds to the triglyceride plasma levels of humans under parenteral nutrition [23].

The generation of hydrogen peroxide was used as a marker for the respiratory burst, which is a critical component of the killing function of neutrophils. Stimulation of neutrophils results in activation of the NADPH oxidase on the cell surface via several messengers such as G protein, phospholipase C (PLC), proteinkinase C (PKC) and increase of cytosolic calcium concentration.

Wanten et al. reported that MCT activated leukocytes and distinctly modulated calcium- and PKC-mediated cell signalling significantly different than LCT, structured lipids (SL) and fish oil based lipids [19]. The difference between MCT and SL seems surprising since SL contains MCT fatty acids.

The LCT/MCT lipid emulsion used in the present study differs from the other two emulsions examined for it contains the saturated MCT caprylic (C8:0) and capric acid (C10:0). Therefore, our results suggested that the medium chain fatty acids caprylic and capric acid do not induce a respiratory burst activity in neutrophils or monocytes. Similar results were reported by Wanten et al. [24], who could demonstrate that the long-chained polyunsaturated arachidonic acid (C20:4), saturated palmitic acid (C16:0), stearic acid (C18:0) and arachidic acid (C20:0) and the medium-chain lauric acid (C12:0) and triglycerides (TC6:0 – TC12:0) all induced significant oxygen radical production in neutrophils, while the medium-chained fatty acids caproic (C6:0), caprylic and capric acid were similar to negative controls. Furthermore, the stimulatory effect seems to be more pronounced with increasing carbon chain length and increasing numbers of double bonds [25].

In contrast to our results, Wanten at al. [26] reported that lipid emulsions containing MCT completely mimicked the effects of phorbol myristate acetate on the serum-treated zymosan induced increase of cytosolic calcium. In another study, the same group demonstrated that LCT/MCT accelerated the respiratory burst of isolated human neutrophils in comparison to LCT. The authors concluded that in contrast to LCT, MCT was capable of inducing oxygen radical formation in neutrophils [6].

In an in vitro study using purified neutrophils isolated by sedimentation and centrifugation Buenestado et al. [27] found an increase of respiratory burst of the human neutrophils after incubation with LCT/MCT. In the same study LCT-MUFA and LCT did not alter the N-formyl-methionyl-leucyl-phenylalanine (fMLP) induced respiratory burst measured by a chemiluminescence method.

Tanaka et al. [28] showed in a cell-free preparation from porcine neutrophils that saturated lauric acid, monounsaturated oleic acid and polyunsaturated arachidonic acid also activated the O_2_^-^-generating system of neutrophils. Furthermore, they demonstrated that the fatty acids themselves and not their metabolites are responsible for the activation, and that the fatty acid must remain in the membrane to maintain the activated state of the O_2_^- ^generating system. In accordance we found LCT-MUFA, that contains high amounts of oleic acid, induces a significant hydrogen peroxide production in neutrophils and monocytes.

For the first time Wanten et al. performed an in vitro study with whole blood incubation and found these results compatible with leukocyte activation [14]. These study analyzed membrane surface markers of adhesion and degranulation on neutrophils and monocytes.

The contradicting reports regarding the in vitro influence of various lipids on the neutrophils oxidative metabolism may be due to the variety of techniques utilized. Additionally, Wanten et al. pointed out that the type of stimulus and the mode of lipid administration, in vivo and ex vivo incubation respectively, strongly determine study outcomes [29].

The multiparameter flow cytometry technique used in the present study has advantages compared to the chemiluminescence, the nitroblue tetrazolium reduction and the cytochrome C reduction techniques [30]. Furthermore, Hatanaka et al. [31] demonstrated that luminol enhanced chemiluminescence is not an appropriate technique to measure reactive oxygen species release by neutrophils treated with fatty acids. Therefore we used the very sensitive protocol described by Rothe et al. [15,32]. This flow cytometry technique is capable of measuring the intracellular activity of the respiratory burst of one selected cell population. The advantage of the whole blood assay used in this study is that both neutrophils and monocytes are left in their physiological environment. We previously demonstrated that the percentage of neutrophils in whole blood generating hydrogen peroxides in response to fMLP was significantly lower (2.5 ± 0.7 %; mean ± SEM) than that in Ficoll isolated cell suspensions (15.1 ± 1.7 %) [12].

## Conclusion

Our findings indicate a significant difference of hydrogen peroxide production by human neutrophils and monocytes after incubation with LCT, LCT-MUFA or LCT/MCT. The most potent respiratory burst stimulus was LCT followed by LCT-MUFA in a dose dependent manner. LCT contains higher amounts of linoleic acid (C18:2) than the other formulations. Oleic acid (C18:1) dominates in LCT-MUFA more than in LCT. We concluded that parenteral nutrition containing these unsaturated fatty acids can induce respiratory burst of neutrophils and monocytes, resulting in an elevated risk of tissue damage by the uncontrolled production of reactive oxygen species. Contradictory observations reported in previous studies may in part be the result of different methods used to determine hydrogen peroxide production.

## Non-financial competing interests

The authors certify that they have no affiliation with or financial involvement in any organization or entity with a direct financial interest in the subject matter or materials discussed in the manuscript.

## Authors' contributions

BJ and DS conceived of the study, performed the statistical analysis and drafted the manuscript. JK carried out the samples analyses. AO participated in the design of the study and helped to draft the manuscript. SC, LW and CW carried out the studies, and participated in its design and helped to draft the manuscript. All authors read and approved the final manuscript.

## Supplementary Material

Additional file 1 Table 1 : Composition and characteristics of lipid emulsions.    Table 2 : Effects of lipid emulsions on percentage of neutrophils having produced hydrogen peroxide.    Table 3 :  Effects of lipid emulsions on percentage of monocytes having produced hydrogen peroxide.
