# Supplementary material for: Unsaturated long-chain fatty acids induce the respiratory burst of human neutrophils and monocytes in whole blood
Source: Nutr Metab (Lond). 2008 Jul 14;5:19. doi: 10.1186/1743-7075-5-19 (PMC2483276; doi:10.1186/1743-7075-5-19)
Supplement: Additional file 1 — Table 1 : Composition and characteristics of lipid emulsions. Table 2 : Effects of lipid emulsions on percentage of neutrophils having produced hydrogen peroxide. Table 3 : Effects of lipid emulsions on percentage of monocytes having produced hydrogen peroxide. [file 1743-7075-5-19-s1.doc]

## **Tables**

Table 1

**Composition and characteristics** of lipid emulsions

|  | LCT/MCT  Lipofundin® | LCT-MUFA  ClinOleic® | LCT  Intralipid® |
| --- | --- | --- | --- |
| Triglyceride Source | Coconut Oil/ Soya Oil (1:1) | Olive Oil | Soya Oil |
| Fatty acid (% w/w of total) |  |  |  |
| Caproic acid (C6:0) | 0.5 | -- | -- |
| Caprylic acid (C8:0) | 28.5 | -- | -- |
| Capric acid (C10:0) | 19.9 | -- | -- |
| Lauric acid (C12:0) | 1 | -- | -- |
| Myristic acid (C14:0) | -- | 0.2 | -- |
| Palmitic acid (C16:0) | 7.4 | 12.2 | 11 |
| Palmitoleic acid (C16:1) | -- | 1.4 | -- |
| C16:3 acid | -- | 0.3 | -- |
| C16:4 acid | -- | 0.5 | -- |
| Stearic acid (C18:0) | 2 | 2.1 | 4 |
| Oleic acid (C18:1) | 11 | 62.3 | 24 |
| Linoleic acid (C18:2) | 29.1 | 18.7 | 53.4 |
| Linolenic acid (C18:3) | 4.5 | 2.3 | 8 |
| Arachidonic acid (C20:4) | 0.2 | -- | 0.1 |
| mean molecular weight | 634 | 873 | 865 |
| Glycerol (g L^-1^) | 25 | 22.5 | 22 |
| pH | 8.0 | 7.0 – 8.0 | 8.0 |

Data provided by the manufactures.

Table 2

**Effects of lipid emulsions on percentage of neutrophils having produced hydrogen peroxide**

|  |  | LCT/MCT | | | LCT-MUFA | | | LCT | | |
| --- | --- | --- | --- | --- | --- | --- | --- | --- | --- | --- |
| time course | control^a)^ | 0.06 mg ml^-1^ | 0.3 mg ml^-1^ | 0.6 mg ml^-1^ | 0.06 mg ml^-1^ | 0.3 mg ml^-1^ | 0.6 mg ml^-1^ | 0.06 mg ml^-1^ | 0.3 mg ml^-1^ | 0.6 mg ml^-1^ |
| 10 min | 5.27±1.32 | 8.36±0.94 | 10.03±1.50 | 9.93±1.60 | 12.24±2.19 | 11.55±1.43* | 13.38±3.05 | 13.00±1.79* | 13.47±1.41* | 15.29±2.67* |
| 20 min | 6.16±0.78 | 9.37±1.08 | 9.60±1.77 | 10.14±1.58 | 9.82±1.49 | 12.76±2.46 | 13.47±1.99* | 13.15±1.77* | 14.47±1.97* | 14.79±1.70* |
| 30 min | 6.50±0.33 | 9.08±1.40 | 9.40±1.22 | 8.91±1.50 | 10.74±2.75 | 11.53±1.19* | 14.66±5.20 | 12.11±1.36* | 13.94±2.41* | 15.54±2.30* |
| 40 min | 7.41±1.10 | 7.45±0.97 | 9.43±1.55 | 8.22±1.14 | 12.28±2.49 | 12.49±2.99 | 15.71±4.55 | 10.56±1.63 | 13.91±1.74* | 16.04±3.06 |
| 50 min | 8.68±1.09 | 9.86±1.23 | 9.66±1.12 | 9.03±1.40 | 12.03±1.94 | 12.64±3.61 | 15.87±3.92 | 13.92±1.99 | 11.87±1.50 | 17.69±3.13 |
| 60 min | 8.14±1.15 | 11.39±1.64 | 8.52±1.57 | 12.1±2.02 | 11.97±2.36 | 16.32±3.66 | 16.25±4.61 | 14.17±2.74 | 17.94±3.30 | 19.56±3.57* |

^a)^negative control with a equal volume of PBS instead of lipid emulsions
*p<0.05 lipid emulsion vs. negative control, Dunnett-t-test; values are expressed as percentage of active cells (mean ± SEM)

Table 3

**Effects of lipid emulsions on percentage of monocytes having produced hydrogen peroxide**

|  |  | LCT/MCT | | | LCT-MUFA | | | LCT | | |
| --- | --- | --- | --- | --- | --- | --- | --- | --- | --- | --- |
| time course | control^a)^ | 0.06 mg ml^-1^ | 0.3 mg ml^-1^ | 0.6 mg ml^-1^ | 0.06 mg ml^-1^ | 0.3 mg ml^-1^ | 0.6 mg ml^-1^ | 0.06 mg ml^-1^ | 0.3 mg ml^-1^ | 0.6 mg ml^-1^ |
| 10 min | 3.55±1.06 | 5.43±0.66 | 4.75±0.71 | 5.56±0.89 | 7.57±1.26 | 7.37±1.47 | 8.99±2.72 | 7.28±1.19 | 7.27±0.87 | 10.40±1.47* |
| 20 min | 4.50±1.07 | 6.29±0.90 | 5.83±1.15 | 5.50±0.78 | 7.03±1.19 | 8.98±2.17 | 10.16±2.21 | 7.31±1.19 | 8.64±1.30 | 11.56±1.39^§^ |
| 30 min | 4.08±0.16 | 6.70±1.48 | 6.82±1.55 | 5.88±1.06 | 7.71±1.47 | 8.92±1.61 | 14.94±6.21 | 7.15±0.93* | 8.44±1.31* | 13.11±1.91^§^ |
| 40 min | 4.71±0.80 | 5.55±1.00 | 7.33±1.79 | 5.85±1.21 | 8.45±1.44 | 10.18±2.27 | 13.48±3.96 | 7.16±1.01 | 8.80±0.98* | 11.66±1.59^§^ |
| 50 min | 5.43±0.65 | 7.30±1.04 | 7.20±1.52 | 7.27±1.43 | 8.52±1.25 | 10.23±3.46 | 12.84±2.77 | 8.03±0.98 | 7.85±0.93 | 14.32±2.40* |
| 60 min | 5.80±1.24 | 9.81±1.64 | 8.01±2.12 | 8.93±2.15 | 8.15±1.27 | 12.46±2.95 | 13.48±3.55 | 8.75±1.14 | 11.54±1.73 | 14.10±1.50* |

^a)^negative control with a equal volume of PBS instead of lipid emulsions
*p<0.05 lipid emulsion vs. negative control, ^§^p<0.05 LCT vs. control and LCT/MCT, Dunnett-t-test; values are expressed as percentage of active cells (mean ± SEM)
